# 

**Published:** 1891-10-17

**Authors:** 


					hHOMAS'S'
iSaoir Westers Infirm art
WITH EXTRA NURSING SUPPLEMENT.
Saturday, October 17th, 1891
wows,
1 tnn IMC A KIT? \ '
* INFANTS
JB
ISINGLASS
AND INVALIDS.

				

